# Dynamics of Maize Carbon Contribution to Soil Organic Carbon in Association with Soil Type and Fertility Level

**DOI:** 10.1371/journal.pone.0120825

**Published:** 2015-03-16

**Authors:** Jiubo Pei, Hui Li, Shuangyi Li, Tingting An, John Farmer, Shifeng Fu, Jingkuan Wang

**Affiliations:** College of Land and Environment, Shenyang Agricultural University, the Ministry of Agriculture Key Laboratory of Northeast Cultivated Land Conservation, Shenyang, Liaoning Province, China; Chinese Academy of Sciences, CHINA

## Abstract

Soil type and fertility level influence straw carbon dynamics in the agroecosystems. However, there is a limited understanding of the dynamic processes of straw-derived and soil-derived carbon and the influence of the addition of straw carbon on soil-derived organic carbon in different soils associated with different fertility levels. In this study, we applied the in-situ carborundum tube method and ^13^C-labeled maize straw (with and without maize straw) at two cropland (Phaeozem and Luvisol soils) experimental sites in northeast China to quantify the dynamics of maize-derived and soil-derived carbon in soils associated with high and low fertility, and to examine how the addition of maize carbon influences soil-derived organic carbon and the interactions of soil type and fertility level with maize-derived and soil-derived carbon. We found that, on average, the contributions of maize-derived carbon to total organic carbon in maize-soil systems during the experimental period were differentiated among low fertility Luvisol (from 62.82% to 42.90), high fertility Luvisol (from 53.15% to 30.00%), low fertility Phaeozem (from 58.69% to 36.29%) and high fertility Phaeozem (from 41.06% to 16.60%). Furthermore, the addition of maize carbon significantly decreased the remaining soil-derived organic carbon in low and high fertility Luvisols and low fertility Phaeozem before two months. However, the increasing differences in soil-derived organic carbon between both soils with and without maize straw after two months suggested that maize-derived carbon was incorporated into soil-derived organic carbon, thereby potentially offsetting the loss of soil-derived organic carbon. These results suggested that Phaeozem and high fertility level soils would fix more maize carbon over time and thus were more beneficial for protecting soil-derived organic carbon from maize carbon decomposition.

## Introduction

Soil organic carbon (SOC) is important to cropland soil, and it influences carbon balance and soil fertility in agroecosystems [1–2]. The difference in SOC content in cropland soils varies by different soil types due to different formation processes and nutrient conditions [3] and by different agricultural management practices. The application of organic material (e.g., crop residues) is one of the major sustainable management practices of cropland soils, and it increases or reduces the amounts of carbon inputs or outputs; improves soil physical, chemical and biological properties; potentially accomplishes the restoration of SOC and improves soil fertility [4–7]. It is also important for carbon transformation for conventional agriculture cropping systems in northeast China by transferring organic matter and nutrients to the soil [8–11]. Most studies in this area reveal that all of the tested organic materials show a gradual decreasing trend over time even though there are decomposition differences among these organic materials [12–14]. However, few studies have compared organic material decomposition between different soil types or different soil fertility levels [15–17]; even fewer studies have quantified the process of external carbon transformation and its contribution to SOC in different cropland soils associated with different fertility levels to examine the interactions of soil type and soil fertility level with external carbon transformation.

Phaeozem and Luvisol [18] are the major high productivity cropland soils in northeast China. Maize residue (*Zea mays L*.) is one of the major external organic materials in this area. Generally, the decomposition of maize residue is a biologically driven process in which a greater portion of residual carbon is oxidized by soil microbes and converted into CO_2_ which is released into the air [19]. Moreover, residual carbon is either incorporated into microbial biomass or transformed into relatively stable humic substances [20]. Both are considered as semi-decomposed or decomposed organic carbon added to SOC [3]. The traditional manner of tracing the decomposition of maize residue is limited to accurately separating the carbon source in a maize-soil system [21]. Nevertheless, the carbon isotopic technique as a diagnostic method is applied throughout a wide range of studies in soil science, agriculture, ecology and biology to make it possible to accurately trace the decomposition of added crop residues [22]. Meanwhile, stable ^13^C is well suited to the study of soil carbon dynamics over time due to its lack of radioactivity [21]. Some laboratory incubation studies use the natural abundance of ^13^C to investigate carbon turnover from C_3_ to C_4_ plant in soil [10, 23–24], while others use enriched ^13^C-labeled plant residue (e.g., maize or rice residues) to explore the fate of carbon in crop-soil ecosystems and the dynamics of residue decomposition in soil [21, 25–27]. However, fewer studies focus on the interactions of cropland soil type and fertility level on crop residue-derived and soil-derived carbon transformation.

Based on the above, the objective of this study is to conduct in-situ experiments by means of a carborundum tube method and ^13^C-labeled maize straw to (1) trace and quantify maize-derived and soil-derived carbon transformation and their contributions to total organic carbon between two cropland soils (i.e., Phaeozem and Luvisol) and between two soil fertility levels (i.e., high and low fertility levels), (2) examine the effects of the dynamics of maize-derived carbon on soil-derived carbon, and (3) investigate the interactions among time, soil type and fertility level on the dynamics of both maize-derived and soil-derived carbon.

## Materials and Methods

### Ethics statement

All necessary permits were obtained from both study sites (i.e., the Key Observation Experiment Station of Gongzhuling Black Soil Ecology and Environment, Jilin Institute of Agricultural Sciences and the Brown Earth Long-Term Experiment Station, Shenyang Agricultural University, China.) for the in-situ field research described and results reported.

### Study sites

**Fig 1:**
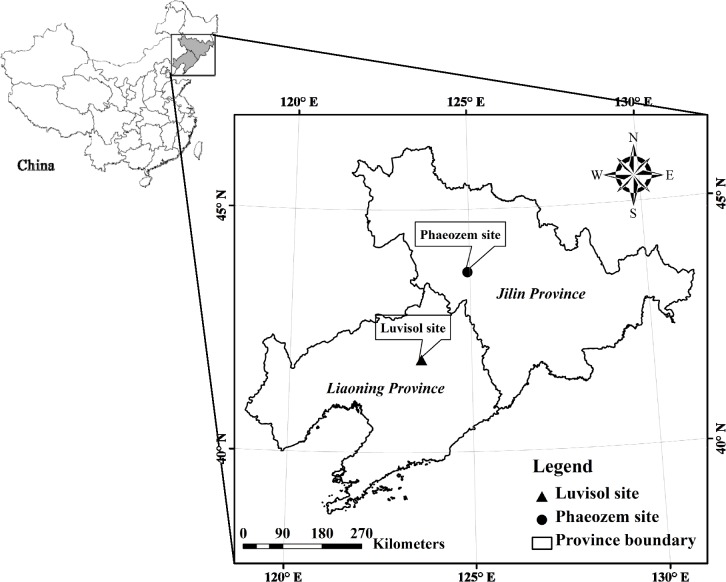
Locations of study sites.

Two long-term cropland experimental sites established on two conventional maize cropping fields were used in this study. A Phaeozem (clay loam, Black soil in Chinese Soil Classification) site was established in 1980 in Jilin Province (43.58°N, 124.77°E, elevation of 220 m), and a Luvisol (loam, Brown earth in Chinese Soil Classification) site was established in 1987 in Liaoning Province (41.82°N, 123.57°E, elevation of 75 m) in northeast China (Fig. 1). The soils on both sites originated from quaternary loess-like sediment without a hydrochloric acid (3 M HCl) reaction in the whole soil body. Both sites belong to a temperate, continental monsoon climate with a mean annual temperature of 4.5°C (Phaeozem site) and 7.6°C (Luvisol site) and have an annual precipitation of 525 mm (Phaeozem site) and 730 mm (Luvisol site). The plot area was 60 m^2^ and 69 m^2^ in Phaeozem and Luvisol sites, respectively.

### Experimental design

The tested soil samples were collected from the topsoil (0–20 cm) of both sites in October 2011 after harvest with three replications. High fertility Phaeozem soil (H-PH) was collected from the plot with an annual application of manure (pig and cow compost, 172.5 kg N ha^−1^ a^−1^) combined with inorganic nitrogen (urea, 75 kg N ha^−1^ y^−1^), phosphorous (diammonium phosphate, 123.75 kg P ha^−1^ y^−1^) and potassium (potassium sulfate, 123.75 kg k ha^−1^ y^−1^), and low fertility Phaeozem soil (L-PH) was collected from the plot without any fertilizer. Moreover, high fertility Luvisol soil (H-LV) was collected from the plot with an annual application of manure (pig compost, 270 kg N ha^−1^ y^−1^), and low fertility Luvisol soil (L-LV) was collected from the plot without any fertilizer. After being air dried, these samples were ground and passed through a 2 mm sieve. Sequentially, a small portion of the samples was continually ground to determine the total organic carbon content and δ^13^C signature, while others were prepared for the in-situ experiment. The basic properties of the soils tested are provided in Table 1.

**Table 1:** The basic properties of tested soils associated with different fertility levels in 2011.

Soil Type	FertilityLevel	TOC (g kg^-1^)	TN (g kg^-1^)	δ^13^C (‰)	MBC (mg kg^-1^)	C/N ratio	pH (H_2_O)	Sand (%)	Silt (%)	Clay (%)
**Phaeozem**	High	29.88a	2.95a	−19.08a	114.59a	10.13a	6.92a	38.07a	32.99a	28.94a
	Low	14.67b	1.39b	−19.28b	70.59b	10.58a	7.01b	38.82a	30.14b	31.04ac
**Luvisol**	High	18.35c	2.00c	−18.82c	92.41c	9.18b	6.39c	27.62b	47.45c	24.93b
	Low	12.33d	1.18d	−18.47d	72.81d	10.49a	6.44c	29.62b	44.90c	25.48c

TOC, total organic carbon; TN, total nitrogen; MBC, microbial biomass carbon; C/N ratio, the ratio of TOC and TN. Different letters in a column indicate significant differences with three replications (*P<0*.*05*).

In addition, enriched ^13^C-labeled maize straw obtained from a ^13^C-pulse labeling experiment in the Luvisol site after harvest in October 2011 was used in this study [26]. The properties of ^13^C-labeled maize straw were: 416.26 g kg^−1^ total carbon, 12.53 g kg^−1^ total nitrogen and 161.72 ‰ δ^13^C signature.

The carborundum tube method [28] combined with ^13^C technology used in this study will help to trace and quantify the transformation of maize-derived carbon and soil-derived carbon in total organic carbon. The carborundum tube has capillary pores with the following dimensions: 38-mm inner diameter, 155-mm height, 8.5-mm tube wall thickness and 140 μm by 70 μm pore size where only water molecules, dissolved organic C and air could penetrate the wall while blocking out crop roots.

The treatments with and without maize straw in both soils associated with high and low fertility levels were arranged in corresponding plots with three replications. For the treatment with maize straw, dry maize straw (5% of added oven dry soil weight, ground to pass through a 0.425 mm sieve) was added to uniformly mix with the air-dried soil sample (100 g oven dry soil weight, passed through a 2 mm sieve) in each carborundum tube, followed by soil water content modified to a field capacity of 70% using distilled water. Then, all carborundum tubes had a lid placed on top and sealed with a tape. A similar procedure was used in the treatment without maize straw. Subsequently, all carborundum tubes were sprayed with a 10 ml solution mixed with local soil and water for acclimation to the local soil environment [28] and then vertically buried to a 5–20 cm depth in the corresponding experimental plot before sowing in early May 2012. The sampling dates are provided in Table 2. On the sampling day, carborundum tubes were collected from each corresponding plot. After removal from the tubes, the soil samples were air dried and ground to determine the total organic carbon content and δ^13^C signature.

**Table 2:** The sampling date and environment situation of the experimental sites.

Site	Experimentalperiod (month)	SamplingDate	Season	Temperatureª(°C)
**Phaeozem**	0	2012.5.3^b^	Spring	16.5
	2	2012.7.9	Summer	26
	6	2012.10.19	Autumn	7
	-	2012.11–2013.3	Winter	< 0
	12	2013.5.6	Spring	18.5
**Luvisol**	0	2012.5.1^b^	Spring	17
	2	2012.7.7	Summer	26.5
	6	2012.10.20	Autumn	8.5
	-	2012.11–2013.3	Winter	< 0
	12	2013.5.4	Spring	17.5

ª, soil surface temperature.

^b^, the date of burying carbonrundum tubes.

Organic carbon content, total nitrogen and δ^13^C signature of soil samples and maize straw were determined using EA-IRMS (Elementar vario PYRO cube coupled to IsoPrime100 Isotope Ratio Mass Spectrometer, Germany). Carbon isotopic composition in this study is reported in δ notation (in ‰ units), relative to the international standard Pee Dee Belemnite (PDB) [22].

### Calculations and statistics

The proportions of maize-derived carbon (*f*_*M*_, %) and soil-derived organic carbon (*f*_*S*_, %) in the treatment with maize straw were estimated by Eq. (1) and Eq. (2) [10, 22]:

$$f_{M}=(\delta^{13}C_{SM}-\delta^{13}C_{So})/(\delta^{13}C_{Mo}-\delta^{13}C_{So})\times \text{100}$$ (1)

$$f_{S}=1\text{00}-f_{M}$$ (2)

Where *δ* ^*13*^ *C*_*SM*_ represents the δ^13^C value of total organic carbon in the treatment with maize straw at a certain time; *δ* ^13^ *C*_*So*_ represents the δ^13^C value of the initial soil; and *δ* ^13^ *C*_*Mo*_ represents the δ^13^C value of the initial maize straw.

If we assume that there is no reaction at the initial state in the treatment with maize straw, we can calculate the initial proportions of maize-derived carbon (*f*_*Mo*_, %) and soil-derived organic carbon (*f*_*So*_, %) in the treatment with maize straw by Eq. (3) and Eq. (4):

$$f_{Mo}=C_{Mo}/(C_{Mo}+C_{So})\times \text{100}$$ (3)

$$f_{So}=C_{So}/(C_{Mo}+C_{So})\times \text{100}$$ (4)

Where *C*_*Mo*_ (g) and *C*_*So*_ (g) represent the initial total organic carbon amounts of added maize straw and soil, respectively. In this case, we calculate the initial *δ* ^13^ *C*_*SMo*_ by transforming Eq. (1) in terms of the determined *δ* ^13^ *C*_*Mo*_ and *δ* ^13^ *C*_*So*_.

Thus, the remaining rates of total organic carbon, maize-derived carbon and soil-derived organic carbon at a certain time in the treatments with and without maize straw can be expressed as follows:

$$r_{SM}=C_{SM}/C_{SMo}\times \text{100}$$ (5)

$$r_{M}=f_{M}\times C_{SM}/C_{Mo}$$ (6)

$$r_{S}=f_{S}\times C_{SM}/C_{So}$$ (7)

$$r_{S^{\prime }}=C_{S}/C_{So}\times \text{100}$$ (8)

Where *r*_*SM*_ (%) represents the remaining rate of total organic carbon in the treatment with maize straw; *r*_*M*_ (%) and *r*_*S*_ (%) represent the remaining rates of maize-derived carbon and soil-derived organic carbon in the treatment with maize straw, respectively; *C*_*SMo*_ (g) represents the initial total organic carbon amount in the treatment with maize straw; *C*_*SM*_ (g) represents the total organic carbon amount in the treatment with maize straw at a certain time; in addition, for the treatment without maize straw, the remaining rate of soil-derived organic carbon ($r_{S^{\prime }}$, %) is expressed as shown in Eq. (8), in which *C*_*S*_ (g) represents the amount of soil-derived organic carbon in the treatment without maize straw at a certain time. Thus, the difference in soil-derived organic carbon between the treatments with and without maize straw (Δ*C*_*S*_, g C kg^−1^ soil) is expressed as shown in Eq. (9):

$$\Delta C_{S}=\left(f_{S}\times C_{SM}-C_{S}\right)/10$$ (9)

The determination of statistically significant differences between all data obtained were performed via ANOVA with a LSD test using JMP 10 statistical software [29]. The paired comparisons of the means for different treatments were analyzed using Student’s t-test.

## Results

### Dynamics of total organic carbon content and δ13C signature

**Fig 2:**
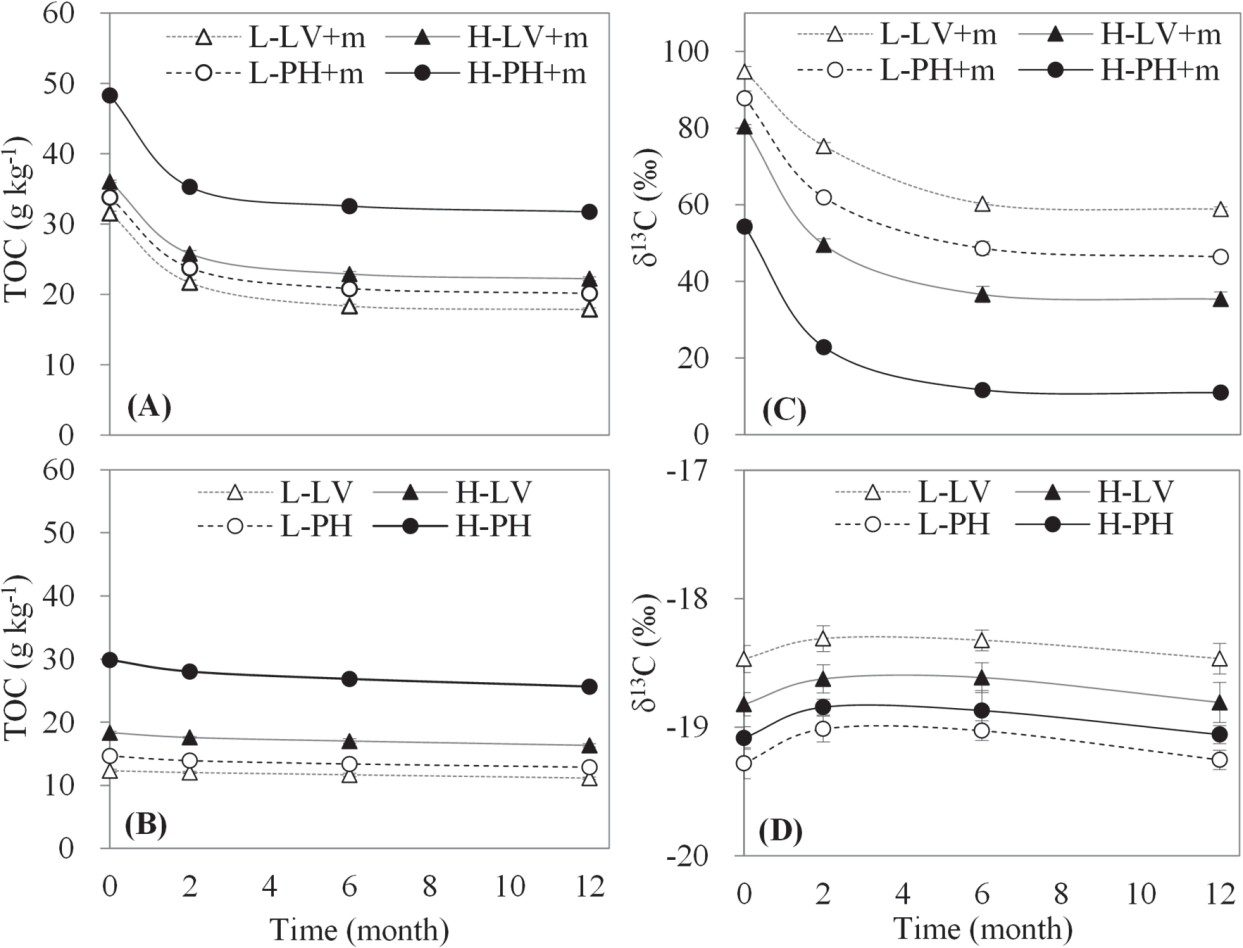
Dynamic patterns of total organic carbon content (TOC) and δ^13^C signature. Data expressed as the means ± SD (n = 3). L-LV+m, low fertility Luvisol plus maize straw; H-LV+m, high fertility Luvisol plus maize straw; L-PH+m, low fertility Phaeozem plus maize straw; H-PH+m, high fertility Phaeozem plus maize straw; L-LV, low fertility Luvisol; H-LV, high fertility Luvisol; L-PH, low fertility Phaeozem; and H-PH, high fertility Phaeozem.

Total organic carbon content and δ^13^C value clearly increased with the addition of maize straw (Fig. 2A, 2C), and were significantly affected by time, soil type, fertility, and maize amendment, (*P<0*.*001*, Table 3-*TOC*, *δ* ^*13*^ *C*). There existed a gradual decline for total organic carbon content and δ^13^C in the treatment with maize straw during the experimental period; meanwhile, a period of rapid decrease occurred during 0 to 2 months (Fig. 2A, 2C). Total organic carbon content in the treatment with maize straw during the experimental period was in the order of high fertility Phaeozem > high fertility Luvisol > low fertility Phaeozem > low fertility Luvisol, while δ^13^C was in the order of low fertility Luvisol > low fertility Phaeozem > high fertility Luvisol > high fertility Phaeozem. Moreover, total organic carbon content and δ^13^C value in the treatment without maize straw during the experimental period varied a little (Fig. 2B, 2D). The order of total organic carbon content also showed that high fertility Phaeozem > high fertility Luvisol > low fertility Phaeozem > low fertility Luvisol, while the δ^13^C followed the order of low fertility Luvisol > high fertility Luvisol > high fertility Phaeozem > low fertility Phaeozem. There were significant interactions between two, three or four factors, but there was no interactions on total organic carbon content between soil type and maize amendment; among time, soil type and maize amendment; among time, fertility and maize amendment; among soil type, fertility and maize amendment; and among four factors (Table 3-*TOC*, *δ* ^*13*^ *C*).

**Table 3:** The ANOVA results of statistical items in this study.

Factor	*df*	*TOC*	*δ* ^13^ *C*	*f*_*M*_	*f*_*S*_	*r*_*SM*_	*r*_*M*_	*r*_*S*_	*r*_*S*_	Δ*C*_*S*_
**T**	3	***	***	***	***	***	***	***	***	***
**S**	1	***	***	***	***	***	***	***	***	***
**F**	1	***	***	***	***	***	***	***	***	***
**M**	1	***	***	-	-	-	-	-	-	-
**T × S**	3	***	*	***	***	***	***	***	***	***
**T × F**	3	***	***	***	***	***	***	***	*	***
**T × M**	3	***	***	-	-	-	-	-	-	-
**S × F**	1	***	***	***	***	NS	***	*	NS	***
**S × M**	1	NS	***	-	-	-	-	-	-	-
**F × M**	1	***	***	-	-	-	-	-	-	-
**T × S × F**	3	***	**	NS	NS	NS	*	NS	NS	*
**T × S × M**	3	NS	*	-	-	-	-	-	-	-
**T × F × M**	3	NS	***	-	-	-	-	-	-	-
**S × F × M**	1	NS	***	-	-	-	-	-	-	-
**T × S × F × M**	3	NS	**	-	-	-	-	-	-	-

*TOC* and *δ* ^*13*^ *C* used 96 samples in total; *f*_*M*_, *f*_*S*_, *r*_*SM*_, *r*_*M*_, *r*_*S*_, $r_{S^{\prime }}$ and Δ*C*_*S*_ used 48 samples in total. T, time; S, soil type; F, Fertility level; M, maize amendment. *TOC*, total organic carbon; *f*_*M*_, the proportion of maize-derived carbon; *f*_*S*_, the proportion of soil-derived organic carbon; *r*_*SM*_, remaining rate of total organic carbon; *r*_*M*_, remaining rate of maize-derived carbon in the treatment with maize straw; *r*_*S*_, remaining rate of soil-derived organic carbon in the treatment with maize straw; $r_{S^{\prime }}$, remaining rate of soil-derived organic carbon in the treatment without maize straw; Δ*C*_*S*_, difference in soil-derived organic carbon between the treatment with and without maize straw. *, **, ***, NS and-, indicate significant differences at *P<0*.*05*, *P<0*.*01*, *P<0*.*001*, no significance and no ANOVA analysis, respectively. *df* indicates degrees of freedom.

### Dynamics of contributions of maize-derived carbon and soil-derived organic carbon to total organic carbon in the treatment with maize straw

**Fig 3:**
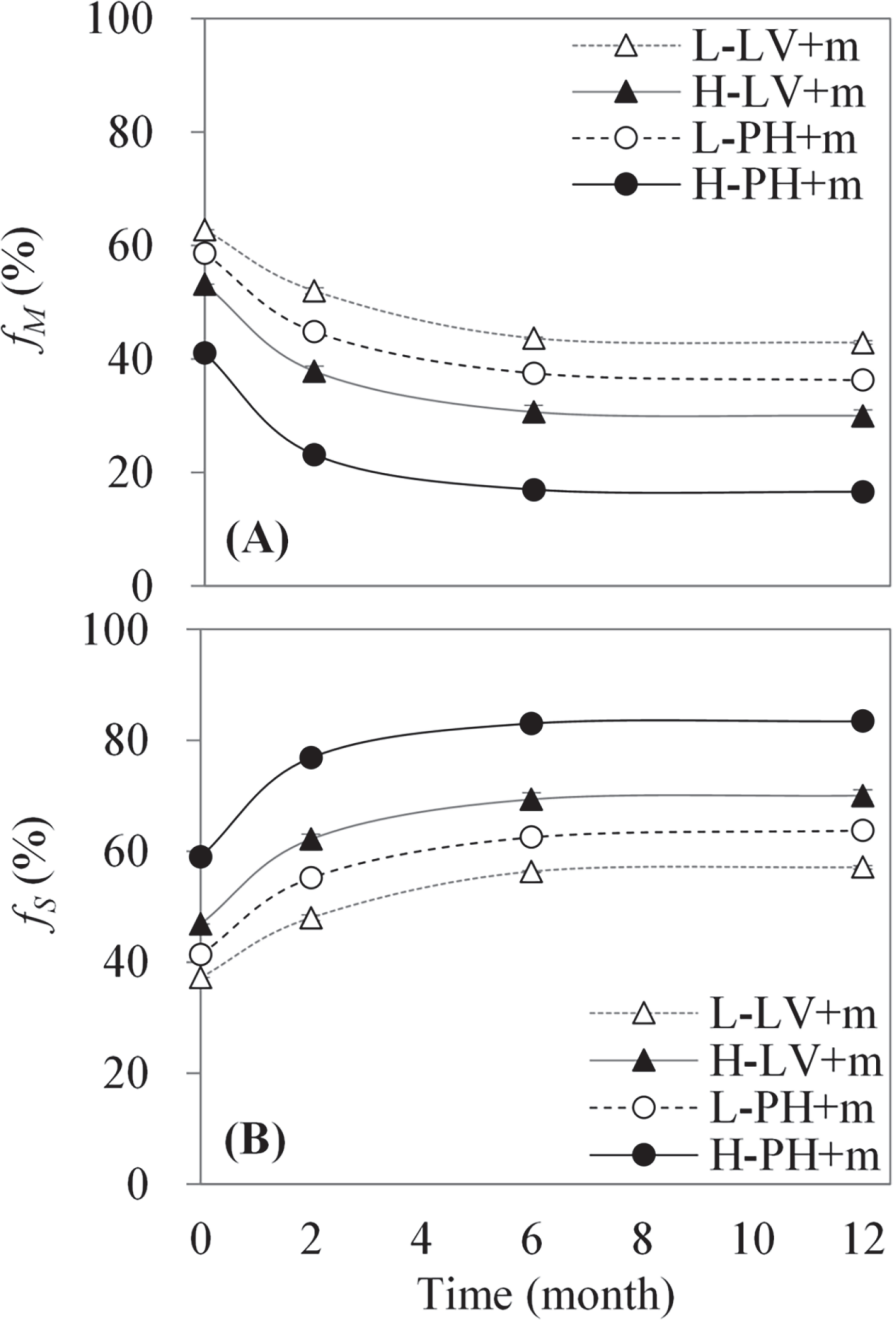
Contributions of maize-derived carbon (A) and soil-derived organic carbon (B) to total organic carbon in the treatment with maize straw. Data expressed as the means ± SD (n = 3). *f*_*M*_, the proportion of maize-derived carbon in total organic carbon; *f*_*S*_, the proportion of soil organic carbon in total organic carbon; L-LV+m, low fertility Luvisol plus maize straw; H-LV+m, high fertility Luvisol plus maize straw; L-PH+m, low fertility Phaeozem plus maize straw; and H-PH+m, high fertility Phaeozem plus maize straw.

The contributions of maize-derived carbon and soil-derived organic carbon to total organic carbon in the treatment with maize straw were opposite in terms of Eq. (2), which showed a gradual decrease in the proportion of maize-derived carbon (Fig. 3A) and a gradual increase in the proportion of soil-derived organic carbon (Fig. 3B) during the experimental period. Both proportions were significantly affected by time, soil type and fertility (*P<0*.*001*, Table 3-*f*_*M*_, *f*_*S*_) in the treatment with maize straw. There were significant interactions between two factors (*P<0*.*001*), while there were no interaction among three factors (Table 3- *f*_*M*_, *f*_*S*_). Additionally, both proportions varied rapidly during 0 to 6 months, especially during 0 to 2 months. Meanwhile, during 0 to 2 months, the contribution of maize-derived carbon to total organic carbon rapidly decreased from 62.82% to 52.03% and from 53.15% to 37.84% on average in the treatments of low and high fertility Luvisol plus maize straw, respectively (Fig. 3A). Similarly, it also rapidly decreased from 58.69% to 44.84% and from 41.06% to 23.17% on average in the treatments of low and high fertility Phaeozem plus maize straw, respectively. In all treatments with maize straw it decreased relatively slowly during 2 to 12 months. At the end of the experimental period, it decreased to 42.90% and 30.00% on average in the treatments of low and high fertility Luvisol plus maize straw, respectively, and to 36.29% and 16.60% on average in low and high fertility Phaeozem plus maize straw, respectively. The overall difference in the proportion of maize-derived carbon in all treatments with maize straw during the experimental period was in the order of low fertility Luvisol > low fertility Phaeozem > high fertility Luvisol > high fertility Phaeozem. Moreover, compared with the trends of maize-derived carbon, all contributions of soil-derived organic carbon showed opposite trends (Fig. 3B).

### Dynamics of the remaining rates of total organic carbon, maize-derived carbon and soil-derived organic carbon

**Fig 4:**
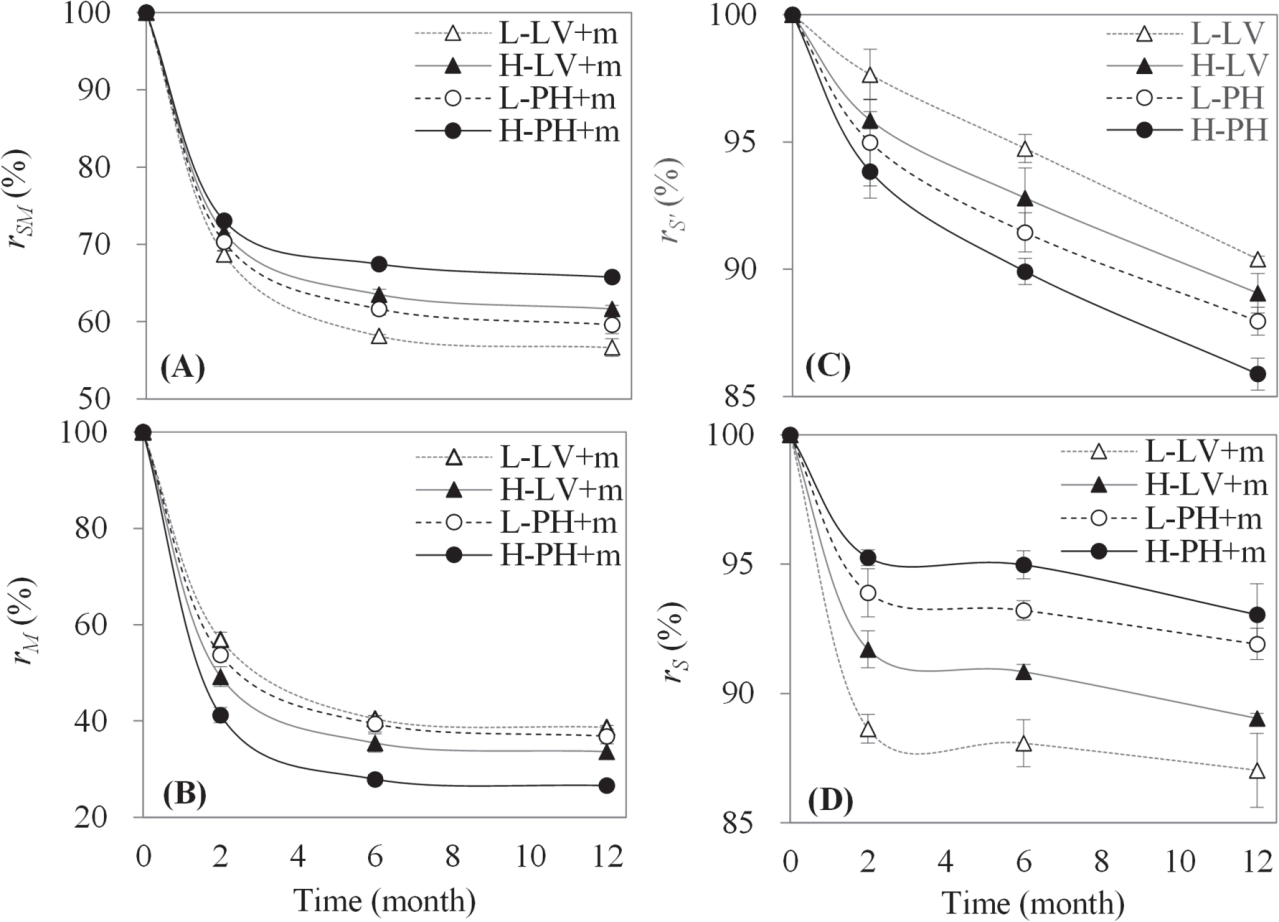
Remaining rates of total organic carbon (A), maize-derived carbon (B), soil-derived organic carbon in the treatments without (C) and with (D) maize straw. Data expressed as the means ± SD (n = 3). *r*_*SM*_, remaining rate of total organic carbon in the treatment with maize straw; *r*_*M*_, remaining rate of maize-derived carbon in the treatment with maize straw;$r_{S^{\prime }}$, remaining rate of soil-derived organic carbon in the treatment without maize straw; *r*_*S*_, remaining rate of soil-derived organic carbon in the treatment with maize straw; L-LV+m, low fertility Luvisol plus maize straw; H-LV+m, high fertility Luvisol plus maize straw; L-PH+m, low fertility Phaeozem plus maize straw; H-PH+m, high fertility Phaeozem plus maize straw; L-LV, low fertility Luvisol; H-LV, high fertility Luvisol; L-PH, low fertility Phaeozem; and H-PH, high fertility Phaeozem.

The remaining rates of total organic carbon, maize-derived carbon and soil-derived organic carbon were significantly affected by time, soil type and fertility, respectively (*P<0*.*001*, Table 3-$r_{SM},r_{M},r_{S},r_{S^{\prime }}$). Meanwhile, there were significant interactions for these remaining rates between two factors, and there were no interactions between soil type and fertility on the remaining rates of total organic carbon in the treatment with maize straw and soil-derived organic carbon in the treatment without maize straw, respectively. Nevertheless, only the remaining rate of maize-derived carbon in the treatment with maize straw had a significant interaction among the three factors (*P<0*.*05*). Moreover, the remaining rates of total organic carbon and maize-derived carbon in the treatment with maize straw significantly decreased during 0 to 6 months, especially during 0 to 2 months (Fig. 4A, 4B), showing an opposite order among these treatments. For the remaining rate of maize-derived carbon, the overall difference among treatments was in the order of low fertility Luvisol > low fertility Phaeozem > high fertility Luvisol > high fertility Phaeozem. At the end of the experimental period, the remaining rates of total organic carbon significantly decreased to 65.76% (high fertility Phaeozem), 61.65% (high fertility Luvisol), 59.59% (low fertility Phaeozem) and 56.65% (low fertility Luvisol) on average, while that of maize-derived carbon significantly decreased to 38.37% (low fertility Luvisol), 36.84% (low fertility Phaeozem), 33.64% (high fertility Luvisol) and 26.59% (high fertility Phaeozem) on average.

In addition, the remaining rates of soil-derived organic carbon in the treatments with and without maize straw (Fig. 4C, 4D) obviously decreased over time, showing a reverse order among these treatments. The remaining rate of soil-derived organic carbon in the treatment with maize straw (Fig. 4D) significantly and rapidly decreased during 0 to 2 months, then slowly decreased during 2 to 6 months and afterwards decreased relatively rapidly again during 6 to 12 months. The overall difference among treatments was meanwhile in the order of high fertility Phaeozem > low fertility Phaeozem > high fertility Luvisol > low fertility Luvisol. At the end of the experimental period, the remaining rates of soil-derived organic carbon in the treatment with maize straw significantly decreased to 93.05% (high fertility Phaeozem), 91.92% (low fertility Phaeozem), 89.04% (high fertility Luvisol) and 87.03% (low fertility Luvisol) on average, while that in the treatment without maize straw decreased to 90.39% (low fertility Luvisol), 89.06% (high fertility Luvisol), 87.96% (low fertility Phaeozem) and 85.88% (high fertility Phaeozem) on average.

### Dynamics of the difference in soil-derived organic carbon between the treatments with and without maize straw

**Fig 5:**
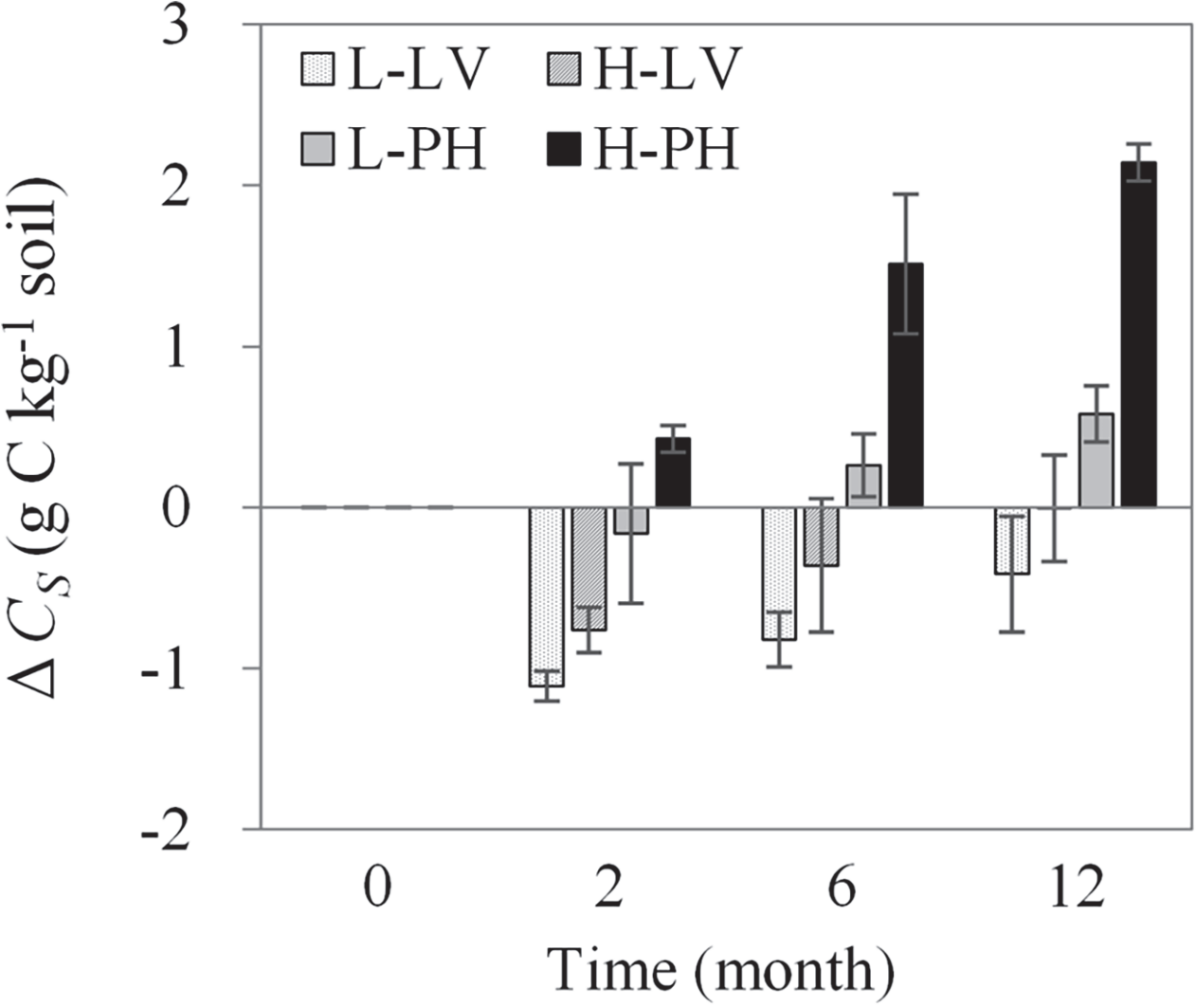
Differences in soil-derived organic carbon between the treatments with and without maize straw. Data expressed as the means ± SD (n = 3). Δ*C*_*S*_, difference of soil-derived organic carbon between the treatment with and without maize straw. L-LV, low fertility Luvisol; H-LV, high fertility Luvisol; L-PH, low fertility Phaeozem; and H-PH, high fertility Phaeozem.

Fertility (*P<0*.*001*, Table 3-Δ*C*_*S*_) significantly increased the difference in soil-derived organic carbon between the treatments with and without maize straw in both soil types (Fig. 5) during the experimental period. Meanwhile, there were significant interactions on the difference in soil-derived organic carbon among two (*P<0*.*001*) or three factors (*P<0*.*05*). The overall pattern of the difference in soil-derived organic carbon between the treatments with and without maize straw was in the order of high fertility Phaeozem > low fertility Phaeozem > high fertility Luvisol > low fertility Luvisol with a trend of an initial decrease followed by an increase in low fertility Phaeozem, high fertility Luvisol and low fertility Luvisol, while high fertility Phaeozem experienced a continuous increase. At the end of the experimental period, the difference in soil-derived organic carbon between the treatments with and without maize straw were 2.14 g C kg^−1^ soil (high fertility Phaeozem), 0.58 g C kg^−1^ soil (low fertility Phaeozem), 0 g C kg^−1^ soil (high fertility Luvisol) and −0.41 g C kg^−1^ soil (low fertility Luvisol) on average.

## Discussion

### Distribution of maize-derived carbon in maize-soil system

**Fig 6:**
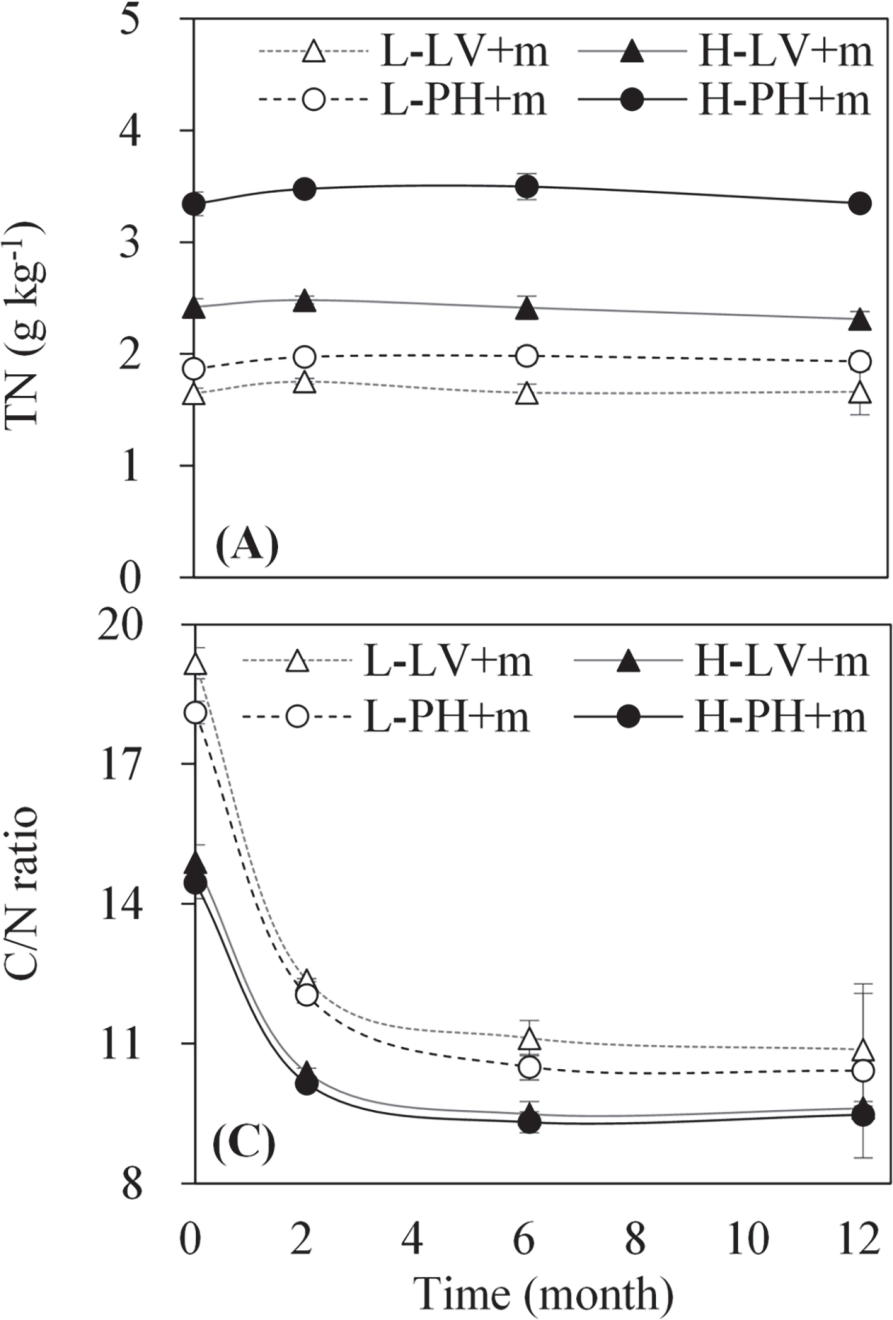
Dynamic patterns of total nitrogen (TN) and C/N ratio in the treatments with maize straw. Data expressed as the means ± SD (n = 3). L-LV+m, low fertility Luvisol plus maize straw; H-LV+m, high fertility Luvisol plus maize straw; L-PH+m, low fertility Phaeozem plus maize straw; and H-PH+m, high fertility Phaeozem plus maize straw.

The addition of maize straw generally increases total organic carbon content, whereas the amount of remaining maize straw decreases over time due to decomposition [14]. Different soil types and fertility levels influence the decomposition and accumulation of maize straw due to the differences in soil characteristics and nutrient conditions [30]. However, an important investigation in this study is the significant interactions of soil type and fertility on the decomposition and accumulation of maize straw (*P<0*.*001*) that were meanwhile influenced by time (*P<0*.*001*) as well. The different patterns of remaining maize-derived carbon among the treatments showed that the decomposition of maize straw fractions was differentiated by soil type and fertility over time. Commonly, the active fractions of maize straw (e.g., sugar, starch, amino acid, protein) are easily and quickly decomposed in the early period (before two months), the slow fractions (e.g., semi-cellulose, cellulose) are decomposed afterwards, and the passive fractions (e.g., lignin and polyphenols) are the last to decompose [13, 24, 31–34]. Our study also found a similar pattern, suggesting that the decomposition of maize straw during 0 to 2 months represented the decomposition of the active fractions of maize straw, followed by the decompositions of the slow fractions (2 to 6 months) and then the passive fractions (6 to 12 months) (Fig. 4B). Based on knowledge on soil microbes, the decomposition of these fractions was mainly controlled by microbial conditions in relation to the different soil types and fertility levels as well [35]. In our case, high microbial activity in Phaeozem and high fertility level soils (Table 1) accelerated the decomposition of maize straw faster than that in Luvisol and low fertility level soils, respectively. Additionally, the results of the C/N ratio in the treatment with maize straw (Fig. 6) suggested that the decomposition of maize-derived carbon was in relation to nitrogen content [17], which further influenced microbial activity that affected the immobilization and mineralization of elements (e.g., carbon and nitrogen) in maize-soil systems [35–36]. In our study, low C/N ratios showed that carbon-limited microbes consume maize or native soil carbon in maize-soil systems [36], which resulted in the differences in decomposition and accumulation of maize straw in different soil types and fertility levels over time.

### Dynamics of soil-derived organic carbon after added maize straw

There is evidence that crop residue carbon input influences the decomposition and accumulation of native soil-derived organic carbon [9–10, 23]. In our study, we observed that the remaining soil-derived organic carbon was significantly differentiated by soil type, fertility and time after the addition of maize straw (Table 3). The addition of maize carbon results in both positive [37] and negative [38] priming effects on soil-derived organic carbon. During the experimental period in this study, the rapid decrease (0 to 2 months) in the remaining rate of soil-derived organic carbon after the addition of maize straw (Fig. 4D) indicated a positive priming effect due to the rapid decomposition of maize straw (Fig. 4C), while the relatively slow decrease during 2 to 6 months indicated a negative priming effect. In this context, the results suggested that active fractions of maize carbon would result in a more positive priming effect on soil-derived organic carbon [31, 39] in the early decomposition period (before two months) of maize straw induced by microbial activity (Table 1) and nutrient conditions (e.g., C/N ratio, Fig. 6) [40]. Moreover, the different nutrient (e.g., microbial activity and nitrogen derived by different soil types and fertility) and hydrothermal conditions over time had interactions with the decomposition and accumulation of soil-derived organic carbon in this study (Table 3). In the early experimental period (0 to 2 months), with the increasing temperature (Table 2) and the decreasing C/N ratio (Fig. 6), the remaining soil-derived organic carbon decreased (Fig. 4). Nevertheless, in the later experimental period (6 to 12 months), a decrease occurred due to the decrease and subsequent increase in temperature (i.e., from autumn to winter then to spring, Table 2), which implied that the freeze-thaw cycle accelerated carbon decomposition in these study sites [41]. The difference in soil-derived organic carbon between soil types and fertility levels in this study suggested that Phaeozem and high fertility level soils were more beneficial for protecting native soil-derived organic carbon from the decomposition of new maize-derived carbon. Additionally, the increase in the difference in soil-derived organic carbon between the treatments with and without maize straw (Fig. 5) demonstrated that maize carbon was incorporated into soil-derived organic carbon, potentially offsetting the loss of soil-derived organic carbon in the later experimental period (after two months), i.e., Phaeozem and high fertility level soils would fix more maize carbon (Fig. 5).

## Conclusions

The results from the in-situ tracing experiment indicated that there existed significant interactions of soil type and fertility over time with the dynamics of maize-derived and soil-derived carbon in a maize-soil system. The contribution of maize-derived carbon to total organic carbon in Phaeozem was higher than in Luvisol, and it significantly decreased with the increasing of fertility level over time. Soil-derived organic carbon, meanwhile, had an opposite trend. The patterns in the amount of remaining maize-derived carbon in the maize-soil system implied that the difference in the decomposition of maize straw fractions during the experimental period was due to different C/N ratios and microbial activities induced by different soil types and fertility levels coupled with hydrothermal conditions. The addition of maize carbon significantly decreased the amount of remaining soil-derived organic carbon in low and high fertility Luvisols, and in low fertility Phaeozem before two months respectively, i.e., positive priming effect; meanwhile, the increasing difference in soil-derived organic carbon between the treatments with and without maize straw after two months showed a negative priming effect, i.e., maize-derived carbon was incorporated into soil-derived organic carbon, thereby offsetting the loss of soil-derived organic carbon. The results at the end of the experimental period suggested that Phaeozem and high fertility level soils would fix more maize straw carbon, i.e., they were more beneficial for protecting native soil-derived organic carbon from the decomposition of new maize-derived carbon.

## References

[1] Lal R. Carbon management in agricultural soils. Mitig. Adapt. Strat. G. 2007; 12, 303–322.

[2] Han FP, Hu W, Zheng JY, Du F, Zhang XC. Estimating soil organic carbon storage and distribution in a catchment of Loess plateau, China. Geoderma. 2010; 154, 261–266.

[3] Brady NC, Weil R. The nature and properties of soils 14th ed. New Jersey: Pearson Education, Inc. Upper Saddle River; 2008.

[4] Follet RF, Paul EA, Leavitt SW, Halvorson AD, Lyon D, Peterson GA. The determination of the soil organic matter pool sizes and dynamics: ^13^C/^12^C ratios of Great Plains soils and in wheat–fallow cropping systems. Soil Sci. Soc.Am. J. 1997; 61, 1068–1077.

[5] Studdert GA, Echeverria HE. Crop rotations and nitrogen fertilization to manage soil organic carbon dynamics. Soil Sci. Soc. Am. J. 2000; 64, 1496–1503.

[6] Leng YH. Effect of long-term fertilization on composition and stability of aggregates in Brown Earth and Black Soil. Ph.D Thesis. University of Shenyang Agricultural University, China. 2008.

[7] Yu S. Effect of long-term different fertilization and plastic mulching on organic fractions and microbial diversity in Brown Earth. Ph.D Thesis. University of Shenyang Agricultural University, China. 2009.

[8] Liang Y, Han XZ, Song C, Li HB. Impacts of returning organic materials on soil labile organic carbon fractions redistribution of mollisol in northeast China. Sci. Agric. Sin. 2011; 44(17), 3565–3574.

[9] Wingeyer AB. Impact of crop and residue management on the physical and chemical stabilization of soil organic matter at farm level. Ph.D Thesis. University of Nebraska-Lincoln. 2011.

[10] Conrad R, Klose M, Yuan Q, Lu YH, Chidthaisong A. Stable carbon isotope fractionation, carbon flux partitioning and priming effects in anoxic soils during methanogenic degradation of straw and soil organic matter. Soil Biol. Biochem. 2012; 49, 193–199.

[11] Casado-Murillo N, Abril A. Decomposition and carbon dynamics of crop residue mixtures in a semiarid long term no-till system: effects on soil organic carbon. Open Agri. J. 2013; 7, 11–21.

[12] Liu M, Zhang L, Yu WT, Shen SM. Decomposition process and residual rate of organic materials C and N in soils. Chin. J. Appl. Ecol. 2004; 18(11), 2503–2506. 18260455

[13] Wang XD, Chen XN, Wang CX, Tian XH, Wu FQ. Decomposition of corn straw in cropland with different fertility. Trans. Chin. Soc. Agric. Eng. 2009; 25(10), 252–257.

[14] Kuang EJ, Chi FQ, Zhang JM, Su QR. Decomposed regularity of organic materials under different condition in black soil. Chin. Agric. Sci. Bull. 2010; 26(7), 152–155.

[15] Xu XC, Zhang JH, Tong GL, Tang YX. Decomposition and remaining rates of organic materials in different soil. Chin. J. Soil Sci. 1985; 1, 21–26.

[16] Dijkstra FA, Cheng WX. Moisture modulates rhizosphere effects on C decomposition in two different soil types. Soil Biol. Biochem. 2007; 39, 2264–2274.

[17] Chen XL, Zhou JB, Liu JL, Gao ZX, Yang XY. Effects of fertilization on carbon/nitrogen ratio of maize straw and its mineralization in soil. Chin. J. Appl. Ecol. 2009; 20(2), 314–319. 19459369

[18] IUSS Working Group WRB. World Reference Base for Soil Resources 2014. International soil classification system for naming soils and creating legends for soil maps. World Soil Resources Reports No. 106. FAO, Rome. 2014.

[19] Thuriès L, Pansu M, Feller C, Herrmann P, Rémy JC. Kinetics of added organic matter decomposition in a Mediterranean sandy soil. Soil Biol. Biochem. 2001; 33, 997–1010.

[20] Adam F, Haider K, Malik KA. Transformation of ^14^C labeled plant components in soil in relation to immobilization-remineralization of N fertilizer. Plant Soil. 1985; 86, 15–25.

[21] Shen QR, Yin SX, Yang CG, Chen W. Application of ^13^C labeling technique to soil science and plant nutrition. Plant Nutr. Fert. Sci. 2000: 6(1), 98–105.

[22] Coleman DC, Fry B. Carbon isotope techniques California, San Diego: Academic Press, Inc. 1991.

[23] Rochette P, Angers DA, Flanagan LB. Maize residue decompositiong measurement using soil surface carbon dioxide fluxes and natural abundance of carbon-13. Soil Sci. Soc. Am. J. 1999; 63, 1385–1396.

[24] Dou S, Zhang JJ, Lichtfouse E, Cao YC. Study on dynamic change of soil orgnaic matter during corn stalk decomposition by δ ^13^C method. Acta Pedo. Sin. 2003; 40(3), 328–334.

[25] Bromand S, Whalen JK, Janzen HH, Schjoerring JK, Ellert BH. A pulse-labelling method to generate ^13^C- enriched plant materials. Plant Soil. 2001; 235, 253–257.

[26] An TT, Schaeffer S, Li SY, Fu SF, Pei JB, Li H, et al Carbon fluxes from plant to soil and dynamics of microbial immobilization under plastic mulching and fertilizer application using ^13^C pulse-labeling. Soil Biol. Biochem. 2015; 80, 53–61.

[27] Comeau LP, Lemke RL, Knight JD, Haughn AB. Carbon input from ^13^C-labeled crops in four soil organic matter fractions. Biol. Fert. Soils. 2013; 49, 1179–1188.

[28] Lin XX, Cheng LL, Xu N, Wen QX. The application of carborundum tube for the determination of decomposition rate of plant residues under field conditions, Acta Pedo. Sin. 1981; 18(1), 97–102.

[29] SAS Institute Inc. JMP 10 basic analysis and graphing, 2nd ed. Cary, NC: SAS Institute Inc. USA 2012

[30] Jiang Y, Zhuang QL, Liang WJ. Soil organic carbon pool and its affecting factors in farmland ecosystem. Chin. J. Ecol. 2007;26(2): 278–285.

[31] Vaieretti MV, Pérez HN, Gurvich DE, Cingolani AM, Cabido M. Decomposition dynamics and physico-chemical leaf quality of abundant species in a montane woodland in central Argentina. Plant Soil. 2005; 278, 205–21.

[32] Juarez S, Nunan N, Duday AC, Pouteau V, Schmidt S, Hapca S, et al Effects of different soil structures on the decomposition of native and added orgnaic carbon. Eur. J. Soil Sci. 2013; 58, 81–90.

[33] Liu S, Chen XN, Wang XD. Changes of the componets and evergy of corn stalk during decomposition process in cropland with different fertility. Afr. J. Agric. Res. 2013; 8(16), 1411–1417.

[34] Pińeiro G, Oesterheld M, Batista WB, Paruelo JM. Opposite changes of whole-soil vs. pools C:N ratios: a case of Simpson’s paradox with implications on nitrogen cycling. Global Change Biol. 2006; 12, 804–809.

[35] Wang LL, Dong M, Zhang L, Du XG. Effects of organic manures with different carbon-to-nitrogen ratios on soil microbial biomass of organic agriculture. Chin. J. Eco-Agric. 2013; 21(9), 1073–1077.

[36] Sylvia DM, Fuhrmann JJ, Hartel PG, Zuberer DA. Principles and applications of soil microbiology, 2nd ed. New Jersey: Inc. Upper Saddle River 2005.

[37] Kuzyakov Y, Friedel JK, Stahr K. Review of mechanisms and quantification of priming effects. Soil Biol. Biochem. 2000; 32, 1483–1498.

[38] Majumder B, Mandal B, Bandyopadhyay PK. Soil organic carbon pools and productivity in relation to nutrient management in a 20-year-old rice-berseem agroecosystem. Biol. Fert. Soils. 2008; 44, 451–461

[39] Werth M, Kuzyakov Y. Assimilate partitioning affects ^13^C fractionation of recently assimilated carbon in maize. Plant Soil. 2006; 284, 319–333.

[40] Rui JP, Peng JJ, Lu YH. Succession of bacterial populations during plant residue decomposition in rice field soil. Appl. Environ. Micro. 2009; 75(14), 4879–4886. doi: 10.1128/AEM.00702-09 19465536 10.1128/AEM.00702-09 PMC2708425

[41] Wang TM, Wang YY, Xiang B, Hui Y, Wang JS. Freeze-thaw cycles effects on humic substances in typical soils in Northeast China. Ecol. Environ. Sci. 2010; 19(12), 2870–2874.

